# Systematic analysis of fatty acid desaturases in breast invasive carcinoma: The prognosis, gene mutation, and tumor immune microenvironment

**DOI:** 10.1097/MD.0000000000038597

**Published:** 2024-06-21

**Authors:** Jie Wang, Qian Zhang, Duanrui Zhou, Yixuan Wang, Huilian Che, Yunjun Ge, Zhangfeng Zhong, Guosheng Wu

**Affiliations:** a Department of Basic Medical Science, Wuxi School of Medicine, Jiangnan University, Wuxi, China; b Macao Centre for Research and Development in Chinese Medicine, State Key Laboratory of Quality Research in Chinese Medicine, Institute of Chinese Medical Sciences, University of Macau, Macao SAR, China; c Jiangnan University Medical Center, Wuxi, China.

**Keywords:** BRCA, *SCD*, *FADS2*, gene mutation, prognosis, tumor immune microenvironment

## Abstract

Breast invasive carcinoma (BRCA) is one of the most common cancers in women, with its malignant progression significantly influenced by intracellular fatty acid (FA) desaturation. Stearoyl-coenzyme A desaturase (SCD) and fatty acid desaturase 2 (FADS2) are two key rate-limiting enzymes that catalyze the FA desaturation process and cooperate to accelerate lipid metabolic activities. In this study, we investigated the potential functions of *SCD* and *FADS2* in BRCA using bioinformatic analysis and experimental validation. The gene expression profiling interactive analysis database showed that the expression of *SCD* or *FADS2* genes was positively linked to worse overall survival and disease-free survival in the Cancer Genome Atlas database-BRCA. The University of Alabama at Birmingham cancer data analysis portal database indicates that the expression and methylation levels of *SCD* or *FADS2* are associated with various clinicopathological factors in patients with BRCA. Moreover, the tumor immune estimation resource and TISCH databases showed a significant positive correlation between the expression of *SCD* and the abundance of CD8+ T cells and macrophage cell infiltration, while the expression of *FADS2* was positively correlated with the abundance of B cells. Meanwhile, *SCD* or *FADS2* had a higher expression in monocytes/macrophages analyzed the BRCA_GSE143423 and BRCA_GSE114727_inDrop datasets. Mechanistically, the Search Tool for the Retrieval of Distant Genes and CancerSEA databases showed that *SCD* and *FADS2* were upregulated in several cell biology signaling pathways, particularly in inflammation, apoptosis, and DNA repair. Finally, *SCD* or *FADS2* knockdown inhibited the proliferation of MCF-7 and MDA-MB-231 cells. In summary, SCD and FADS2 play significant roles in BRCA development, suggesting that they may serve as potential therapeutic targets for BRCA treatment.

## 1. Introduction

Breast invasive carcinoma (BRCA) is the most common cancer in women and a leading cause of cancer-related death worldwide.^[1–3]^ Although chemotherapy, radiotherapy, endocrinology, and immunotherapy have reduced the mortality of patients with BRCA, effective therapeutic strategies are still limited.^[4,5]^ In the 1980s, Ookhtens et al found that almost all ^14^C labeled esterified fatty acids (FAs) are originated from de novo FA synthesis in Ehrlich ascites tumor cells.^[6]^ Subsequent studies have revealed that de novo FA synthesis plays a crucial role in tumorigenesis and progression in various tumor cells, and the inhibition of de novo FA synthesis has been linked to BRCA growth suppression and improved survival rates in vivo and in vitro.^[7–9]^

FA desaturation is the final step in de novo FA synthesis and is at the center of tumor lipid metabolism. To maintain high proliferation, tumor cells need to meet the exuberant lipid demand by synthesizing saturated fatty acids (SFA) and then modify the rigidity of SFA on the cell membrane by converting SFA to monounsaturated fatty acid (MUFA) or polyunsaturated fatty acid.^[10]^ In addition, MUFA and polyunsaturated fatty acid are also important messengers in intercellular signaling.^[11]^ Conversely, accumulation of SFA in cells can cause lipotoxicity in cells and accelerate tumor death.^[12,13]^ Therefore, FA desaturation is closely related to tumor occurrence and development and is the key to determining the fate of tumors.^[14]^

Stearoyl-CoA desaturase (SCD), an enzyme involved in FA desaturation, is crucial for regulating the SFA/MUFA ratio and has been identified as an important regulator of cancer cell survival and progression.^[15]^ Many malignancies, including cancer stem cells and invasive cells, have been reported to exhibit high levels of SCD. Our previous study also demonstrated that SCD was correlated with poor prognosis in BRCA patients, and targeting SCD may be a novel therapeutic strategy for BRCA treatment.^[16]^ However, in 2019, Kim Vriens et al found that SCD inhibition led to the activation of its desaturating isoenzyme fatty acid desaturase 2 (FADS2), which continued to produce MUFAs and promote tumor growth.^[17]^ FADS2, also called acyl-CoA 6-desaturase, is another crucial enzyme in FA desaturation that is responsible for long-chain polyunsaturated fatty acids.^[18–20]^ Increasing evidence suggests that inhibiting FADS2 activity can suppress the growth of various cancers, including melanoma,^[21]^ lung cancer,^[22]^ and ovarian cancer.^[23]^ Furthermore, FADS2 promotes the occurrence and development of breast cancer cells by regulating the epithelial-mesenchymal transition process.^[24]^ Remarkably, knockdown of SCD or FADS2 enhanced the sensitivity of lung cancer and ovarian cancer cells to ferroptosis, which is a type of iron-dependent cell death that provides novel avenues for clinical intervention.^[22,23]^

In this study, we employed multiple bioinformatic methods to explore the relationship between *SCD* or *FADS2* expression and various factors in BRCA, including prognostic value, pathological stages, clinicopathological characteristics, genetic alterations, functional states at the single-cell level, and immune infiltration. Our findings offer new insights into the roles of FA desaturation in BRCA, connect the expression of 2 key desaturases with the BRCA tumor microenvironment (TME), and highlight them as potential therapeutic targets for BRCA treatment.

## 2. Methods

### 2.1. Gene expression profiling interactive analysis

Gene expression profiling interactive analysis (GEPIA, http://gepia.cancer-pku.cn/) consists of RNA sequencing expression data of 9736 tumors and 8587 normal samples derived from the Cancer Genome Atlas (TCGA) and the Genotype-Tissue Expression databases.^[25]^ In this study, GEPIA single-gene analysis was used to analyze the prognosis of *SCD* or *FADS2*. The prognostic values of cancer patients, including overall survival (OS) and disease-free survival (DFS), and survival curves were estimated using the Kaplan–Meier method. Hazard ratios (HR) with 95% confidence intervals are shown in the survival plot, and *P* < 0.05 was considered statistically significant.

### 2.2. PrognoScan

PrognoScan (http://dna00.bio.kyutech.ac.jp/PrognoScan/) is a widely used online database for meta-analysis of the prognostic significance of genes.^[26]^ In this study, PrognoScan was used to analyze the association between *SCD* or *FADS2* expression and survival in patients with breast cancer.

### 2.3. The Tumor Immune Estimation Resource

The Tumor Immune Estimation Resource (TIMER, https://cistrome.shinyapps.io/timer/) is a database for the comprehensive analysis of tumor-infiltrating immune cells,^[27]^ which consists of 10897 samples of 32 cancer types from the TCGA database. In this study, the TIMER database was used to analyze the difference *SCD* or *FADS2* expression between tumor and normal tissues, and to evaluate the correlation between gene expression and abundance of immune infiltrates. It contains 6 immune infiltrates (B cells, CD4+ T cells, CD8+ T cells, neutrophils, macrophages, and dendritic cells) and various gene markers. Gene expression levels were assessed using log_2_ TPM.

### 2.4. TISIDB

TISIDB (http://cis.hku.hk/TISIDB/index.php/) is a database that integrates many heterogeneous data sources to assess interactions between tumors and the immune system.^[28]^ In this study, we explored the relationship between *SCD* or *FADS2* expression and molecular subtypes using pan-cancer analysis, especially in BRCA.

### 2.5. The University of Alabama at the Birmingham Cancer Data Analysis Portal

The University of Alabama at the Birmingham Cancer Data Analysis Portal (UALCAN) (http://ualcan.path.uab.edu/) is a comprehensive web portal for analyzing TCGA data.^[29]^ In this study, the expression and promoter methylation levels of *SCD* and *FADS2* in BRCA were analyzed based on sample type, sex, age, race, nodal metastasis status, histological subtypes, major subclasses, menopause status, and individual cancer stages. The *t*-test was used for analysis, and *P* < 0.05.

### 2.6. cBioPortal

The cBio Cancer Genomics Portal (cBioPortal, http://www.cbioportal.org/) provides data on 10967 tumor samples from 20 cancer studies.^[30,31]^ The samples contained gene mutations, copy number alterations, mRNA expression z-scores, and protein expression z-scores. In our study, the genomic profiles (1084 samples) included genetic alterations, co-expression, and network modulation from the genomic identification of significant targets in cancer, and *SCD* or *FADS2* mRNA expression z-scores relative to all samples with a threshold of ± 2.0.

### 2.7. The tumor immune single-cell hub

The tumor immune single-cell hub (TISCH, http://tisch.comp-genomics.org/) is a single-cell RNA sequencing database focusing on the TME,^[32]^ which can provide detailed cell-type annotation at the single-cell level, enabling exploration of the TME across a diverse range of cancers. We used datasets from BRCA_GSE143423 and BRCA_GSE143423 to analyze *SCD* or *FADS2* expression in BRCA as a single-cell subset.

### 2.8. The Search Tool for the Retrieval of Distant Genes Database

The Search Tool for the Retrieval of Distant Genes Database (STRING, https://string-db.org/) compiles, assesses, and combines publicly available protein–protein interaction (PPI) data and augments them with computational forecasts of potential functions. This study submitted variously expressed *SCD* or *FADS2*-related genes to STRING to obtain information about the top 20 related PPI networks and visualize the protein–protein interactive network.^[33]^

### 2.9. CancerSEA

CancerSEA (http://biocc.hrbmu.edu.cn/CancerSEA/) was created to decode Pearson correlations between 14 different single-cell functional states and relevant genes in human malignancies.^[34]^ In this study, we used CancerSEA to investigate the relationships between *SCD* or *FADS2* functional states and invasive breast carcinoma at the single-cell level.

### 2.10. Cell culture

MCF-7 and MDA-MB-231 cells were obtained from the Cell Bank of the Shanghai Institute for Biological Sciences, Chinese Academy of Sciences. MCF-7 and MDA-MB-231 cells were cultured in Dulbecco modified Eagle medium (Sigma, St. Louis, MO) with 10% fetal bovine serum (HyClone, Logan, UT), 100 U/mL penicillin, and 100 mg/mL streptomycin. cells were maintained at 37 °C in a humidified environment in an incubator with 5% CO_2_.

### 2.11. Small interfering RNA

*SCD* and *FADS2* siRNAs used in this study were purchased from Geenchem (Shanghai, China). Lipofectamine 8000 from Beyotime (Shanghai, China) was used to transfect siRNA into MCF-7 and MDA-MB-231 cells according to the manufacturer’s instructions (siRNA-NC, 5′-UUCUCCGAACGUGUCACGUdTdT-3′ forward, and 5′-ACGUGACACGUUCGGAGAAdTdT-3′ reverse), siRNA-*SCD* (5′-GCACAUCAACUUCACCACATT-3′ forward, and 5′-UGUGGUGAAGUUGAUGUGCTT-3′ reverse), and siRNA-*FADS2* (5′-CCGCAAGGUUUACAACAUCACCAAA-3′ forward, and 5′-UUUGGUGAUGUUGUAAACCUUGCGG-3′ reverse).

### 2.12. Cell viability

Inhibition of proliferation of MCF-7 and MDA-MB-231 cells (5 × 10^3^ cells/well) was monitored by the 3-(4,5-dimetrylthiazol)-2,5-diphenyltetrazolium bromide assay. Exponentially growing cells were seeded into 96-well plates, after incubation with different drugs, MF438 (0–50 μM) and A939572 (0–50 μM) were purchased from MedChem Express (NJ).10 μL 3-(4,5-dimetrylthiazol)-2,5-diphenyltetrazolium bromide (1 mg/mL) solution was added to each well and the plates were incubated for an additional 4 hours. Formazan crystals were dissolved by adding 100 μL of DMSO. The absorbance was measured at 490 nm using a BioTek Synergy H4 all-in-one microplate reader (Vermont).

### 2.13. Western blot

Cell lysis buffer for western blotting and cell lysates (Beyotime, Shanghai, China) containing protease inhibitors were used to extract proteins from cells, and the total protein concentration was determined using a BCA Protein Assay Kit (Bio-Rad, CA). Protein samples were collected for SDS-PAGE and then transferred to PVDF (Bio-Rad, CA). After blocking with 5% nonfat milk, the membranes were treated with primary antibodies (1:1000 dilution) and then secondary antibodies (1:3000 dilution). All antibodies were purchased from Cell Signaling Technology (Danvers, MA). Finally, membrane visualization was performed using an enhanced chemiluminescence system, and protein bands were detected using ChemiDOC MP (Bio-Rad, CA). The density of immunoreactive bands was quantified using ImageJ software (National Institute of Health, Bethesda, MA).

### 2.14. Statistical analysis

All in vitro experiments were repeated at least 3 times, and the results are presented as mean ± standard deviation. Student *t* test was used to test for statistical significance. Data were analyzed using GraphPad Prism 7.0 (GraphPad Software, San Diego, CA). *P* < 0.05 was considered statistically significant.

## 3. Results

### 3.1. FA desaturase genes are potential prognostic markers in BRCA

**Figure 1.**
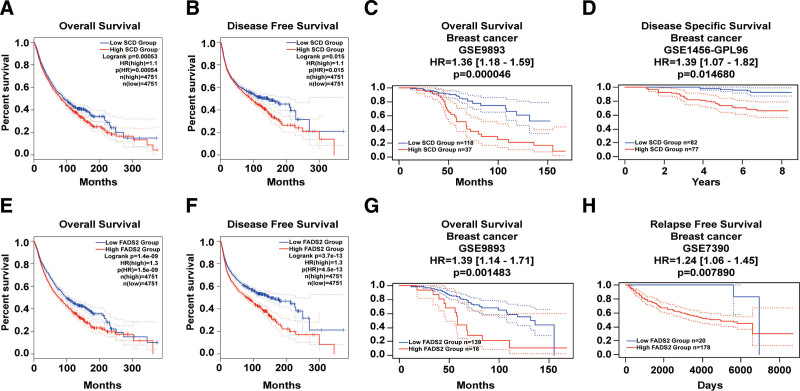
Prognostic analysis of *SCD* or *FADS2* in BRCA. Overall survival analysis of *SCD* (A) or *FADS2* (E) and disease-free survival analysis of *SCD* (B) or *FADS2* (F) in 33 types of human cancers were determined using the GEPIA database. Overall survival analysis of *SCD* (C) or *FADS2* (G), and disease-specific survival analysis of *SCD* (D) or relapse-free survival analysis of *FADS2* (H) in BRCA using the PrognoScan database.

The prognostic ability of *SCD* and *FADS2* expression in pan-cancer was evaluated using GEPIA database. Based on our data, *SCD* expression was positively associated with worse OS (HR = 1.1, log-rank *P* = 0.00054) (Fig. 1A) and DFS (HR = 1.1, log-rank *P* = 0.015) (Fig. 1B). Meanwhile, *FADS2* expression had a stronger positive correlation with worse OS (HR = 1.3, log-rank *P* = 1.5e‐09) (Fig. 1E) and DFS (HR = 1.3, log-rank *P* = 3.7e‐13) compared to *SCD* (Fig. 1F). In addition, the prognostic value of *SCD* or *FADS2* expression in BRCA was investigated using PrognoScan based on 3 different databases (BRCA_GSE9893, BRCA_GSE1456-GPL96, and BRCA_GSE7390), and a negative correlation was found between the overexpression of *SCD* (HR = 1.36 [1.18–1.59], logrank *P* = 0.000046) (Fig. 1C) or *FADS2* (HR = 1.39 [1.14–1.71], log-rank *P* = 0.001483) (Fig. 1G) and OS. *SCD* (HR = 1.39 [1.07–1.82], log rank *P* = 0.014680) (Fig. 1D) or *FADS2* (HR = 1.24 [1.06–1.45], logrank *P* = 0.007890) (Fig. 1H) expression was positively associated with worse disease-specific survival. In general, these results suggest that *SCD* or *FADS2* could be potential prognostic markers in BRCA.

### 3.2. Expression of FA desaturase genes are associated with BRCA molecular subtypes

**Figure 2.**
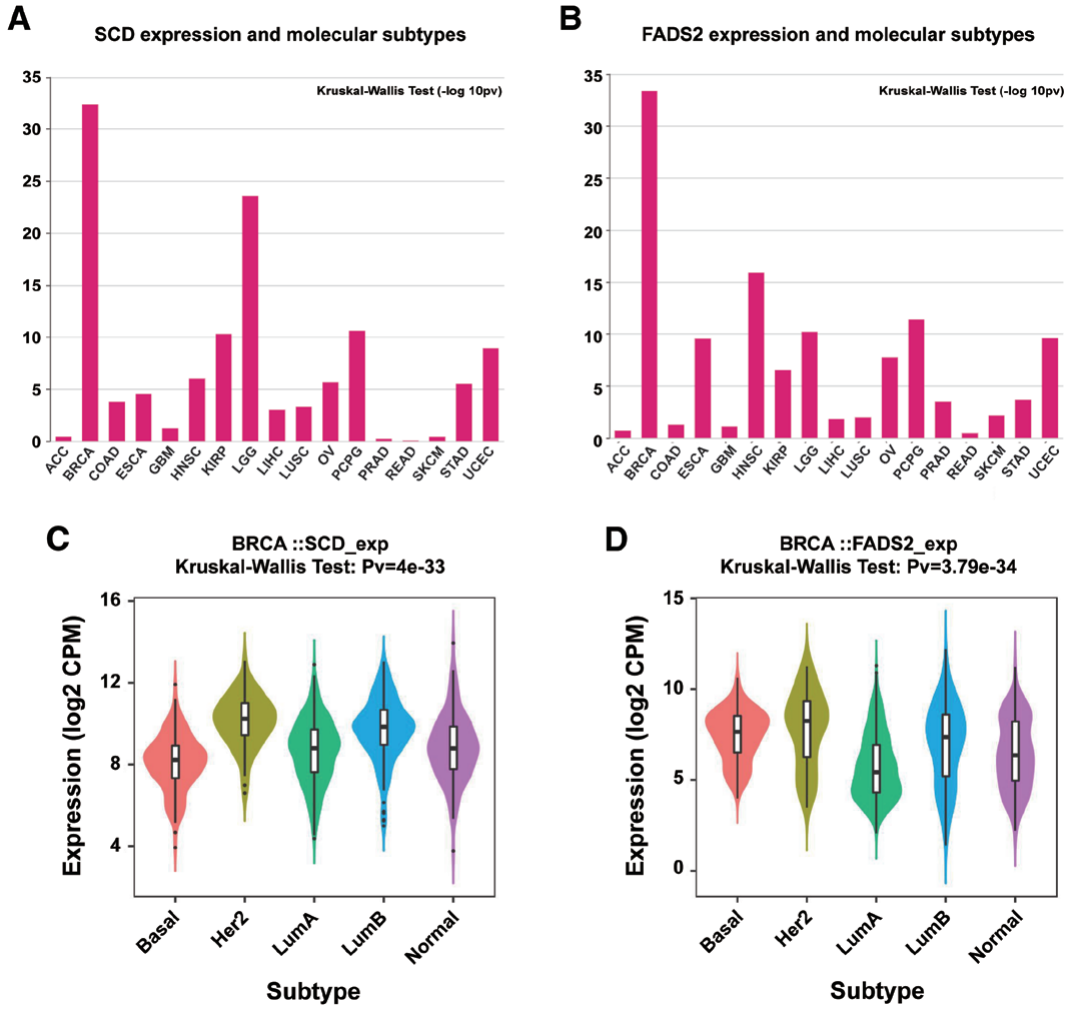
Correlation between *SCD* or *FADS2* expression and molecular subtypes. Association between *SCD* (A) and *FADS2* (B) expression and molecular subtypes in human cancers. The expression of *SCD* (C) or *FADS2* (D) in different molecular subtypes of BRCA using the TISIDB database (n = Basal 172, Her2 73, LumA 508, LumB 191, Normal 137). Her2 = human epidermal growth factor receptor 2, LumA = luminal A, LumB = luminal B.

Based on gene expression profiles and histological classification, BRCA can be categorized into luminal A, luminal B, human epidermal growth factor receptor 2 (Her2), positive and triple-negative breast cancer.^[35–37]^ To further determine the relationship between *SCD* or FADS2 expression and tumor molecular subtypes, we assessed the association between *SCD* (Fig. 2A, Fig. S1, Supplemental Digital Content, http://links.lww.com/MD/M965) or *FADS2* (Fig. 2B, Fig. S2, Supplemental Digital Content, http://links.lww.com/MD/M966) expression and pan-tumor subtypes using the TISIDB database. The results showed that the *SCD* level varied significantly in colon adenocarcinoma, esophageal carcinoma, head and neck squamous cell carcinoma, kidney renal papillary cell carcinoma, brain lower grade glioma, liver hepatocellular carcinoma, uterine corpus endometrial carcinoma, ovarian serous cystadenocarcinoma, pheochromocytoma and paraganglioma, stomach adenocarcinoma, and lung squamous cell carcinoma molecular subtypes and the *FADS2* level also varied significantly in esophageal carcinoma, head and neck squamous cell carcinoma, kidney renal papillary cell carcinoma, lower grade glioma, liver hepatocellular carcinoma, uterine corpus endometrial carcinoma, ovarian serous cystadenocarcinoma, pheochromocytoma and paraganglioma, prostate adenocarcinoma, skin cutaneous melanoma, stomach adenocarcinoma, and lung squamous cell carcinoma molecular subtypes. Based on the molecular subtype classification from the TCGA-BRCA database, *SCD (P*-value = 4e–33) and *FADS2 (P*-value = 3.79e–34) are good predictors of molecular subtypes in BRCA. Specifically, *SCD* was highly expressed in Her2 positive, luminal A, and luminal B subtypes (Fig. 2C), while *FADS2* was highly expressed in the basal, Her2 positive, and luminal B subtypes (Fig. 2D). The correlations between *SCD* or *FADS2* and tumor molecular typing are detailed in Table 1. The results revealed that the expression of *SCD* and FADS2 are related to the molecular subtypes of tumors, especially in BRCA.

**Table 1:** Stearoyl-CoA desaturase 1/fatty acid desaturase 2 sample number of molecular subtypes.

Cancer abbreviation	TCGA cancer type	Total sample number of molecular subtypes	Sample number of molecular subtypes (left to right)	*SCD*/*FADS2* Kruskal–Wallis test: Pv
ACC	Adrenocortical carcinoma	78	19/27/32	3.74e‐01/2.02e‐01
BRCA	Breast invasive carcinoma	1081	172/73/508/191/137	4e‐33/3.79e‐34
COAD	Colon adenocarcinoma	341	226/49/6/60	1.66e‐04/5.2e‐02
ESCA	Esophageal carcinoma	169	74/90/1/2/2	2.85e‐05/2.85e‐10
GBM	Glioblastoma multiforme	119	47/2/5/12/53	5.75e‐02/7.68e‐02
HNSC	Head and neck squamous cell carcinoma	276	67/87/48/74	9.49e‐07/1.33e‐16
KIRP	Kidney renal papillary cell carcinoma	159	95/35/22/9	5.18e‐11/2.9e‐07
LGG	Brain lower grade glioma	511	23/171/234/12/45/26	2.27e‐24/6.34e‐11
LIHC	Liver hepatocellular carcinoma	782	164/255/363	9.97e-04/1.6e-02
LUSC	Lung squamous cell carcinoma	170	42/63/26/39	4.9e-04/1.01e-02
OV	Ovarian serous cystadenocarcinoma	293	66/78/71/78	2.09–06/1.72e-08
PCPG	Pheochromocytoma and paraganglioma	173	22/68/61/22	2.49e‐11/3.78e‐12
PRAD	Prostate adenocarcinoma	333	152/28/14/4/37/9/3/86	5.94e‐01/3.18e‐04
READ	Rectum adenocarcinoma	115	102/9/4/3	8.59e‐01/3.55e‐01
SKCM	Skin cutaneous melanoma	315	150/27/92/46	3.83e‐01/6.77e‐03
STAD	Stomach adenocarcinoma	383	223/30/50/7/73	3.02e‐06/2.15e‐04
UCEC	Uterine corpus endometrial carcinoma	507	160/144/124/79	1.11e‐09/2.58e‐10

### 3.3. Expression of FA desaturase genes correlated with clinicopathological characteristics in BRCA

**Figure 3.**
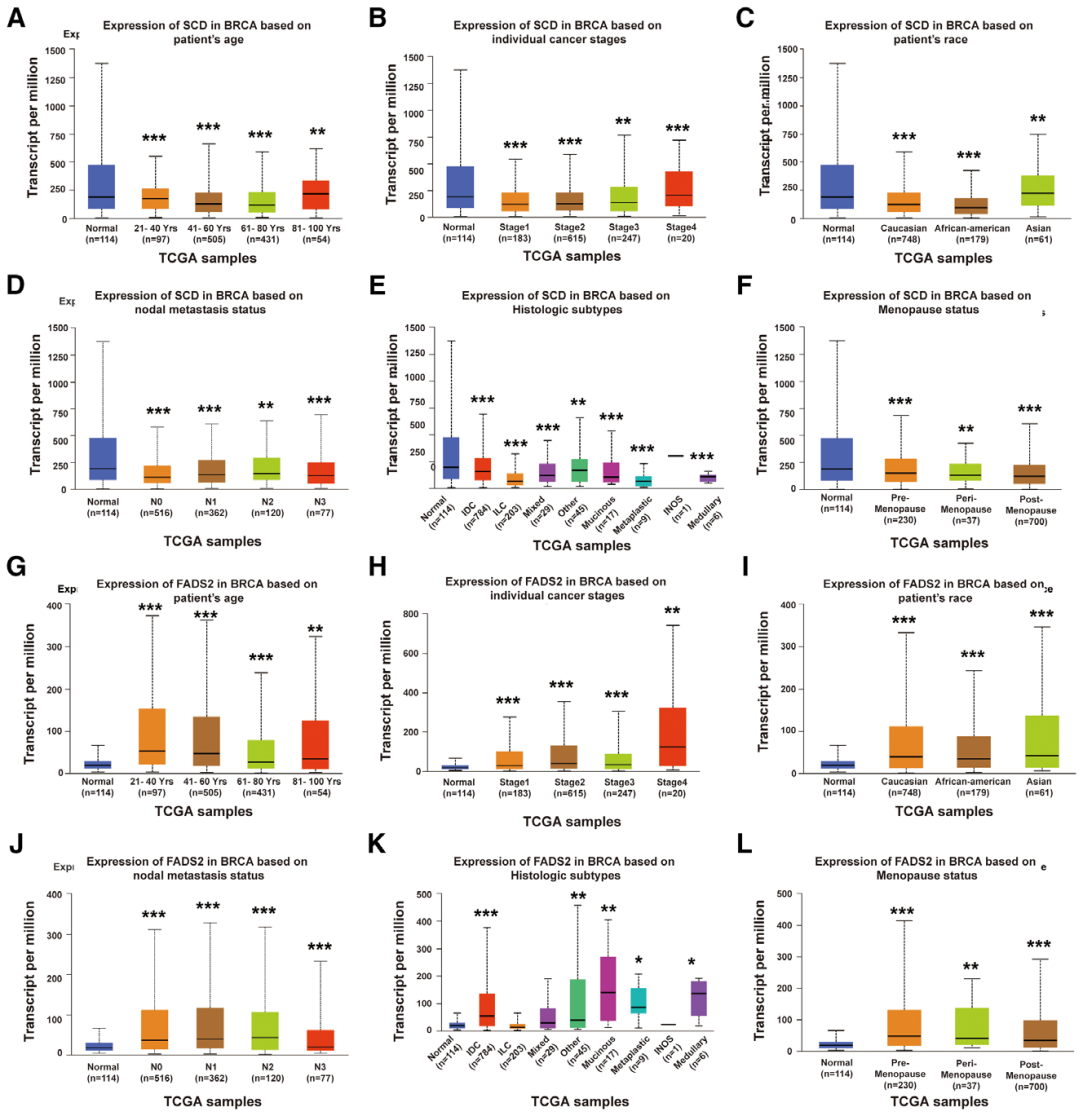
The *SCD* or *FADS2* transcription in different clinical characteristics of patients in BRCA. Box plots evaluating *SCD* expression in BRCA among different groups of patients based on clinical parameters including patient age (A), individual cancer stages (B), patient race (C), nodal metastasis status (D), histological subtypes (E), menopause status (F), as well as *FADS2* expression in BRCA patients based on patient age (G), individual cancer stages (H), patient race (I), nodal metastasis status (J), histological subtypes (K), and menopausal status (L) using the UALCAN database (*P < 0.05; **P < 0.01; ***P < 0.001).

Next, we investigated the correlation between *SCD* or *FADS2* expression and various clinicopathological characteristics based on UALCAN analysis in BRCA samples. The results showed that *SCD* was significantly upregulated in BRCA patients aged 80–100 years (Fig. 3A), in individuals with stage 4 cancer (Fig. 3B), and in the Asian race (Fig. 3C). It was downregulated in nodal metastasis status (Fig. 3D), histological subtypes (Fig. 3E), and menopausal status (Fig. 3F). Additionally, *FADS2* was remarkably upregulated across a wide range of BRCA patient characteristics, including age (21–100 years), all cancer stages (1–4), races (Caucasian, African-American, and Asian), nodal metastasis status (N0–3), histological subtypes (IDC, other, mucinous, metaplastic, and medullary), and menopausal status (premenopause, peri-menopause, and postmenopause) (Fig. 3G–L). Taken together, these results suggest a close correlation between *SCD or FADS2* expression and clinicopathological characteristics in BRCA. These enzymes could serve as diagnostic tumor markers for BRCA, considering with different pathological parameters. Compared to *FADS2, SCD* is more targeted for Asian patients, aged 81–100 years, and with cancer stage 4.

### 3.4. Promoter methylation levels and gene mutations of FA desaturase genes in BRCA

Gene silencing and hypermethylation of tumor DNA are early molecular abnormalities in carcinogenesis. Promoter DNA methylation has been shown to affect transcriptional repression and participates in tumor oncogenesis.^[38]^ Therefore, the promoter methylation levels of *SCD* or *FADS2* were compared, and UALCAN analysis was performed using TCGA-BRCA samples. The results suggested that promoter methylation levels of *SCD* (Fig. S3A, Supplemental Digital Content, http://links.lww.com/MD/M967) and *FADS2* (Fig. S3B, Supplemental Digital Content, http://links.lww.com/MD/M967) were upregulated in other age, race, individual cancer stages, nodal metastasis status, tumor history, and menopause status groups.

**Figure 4.**
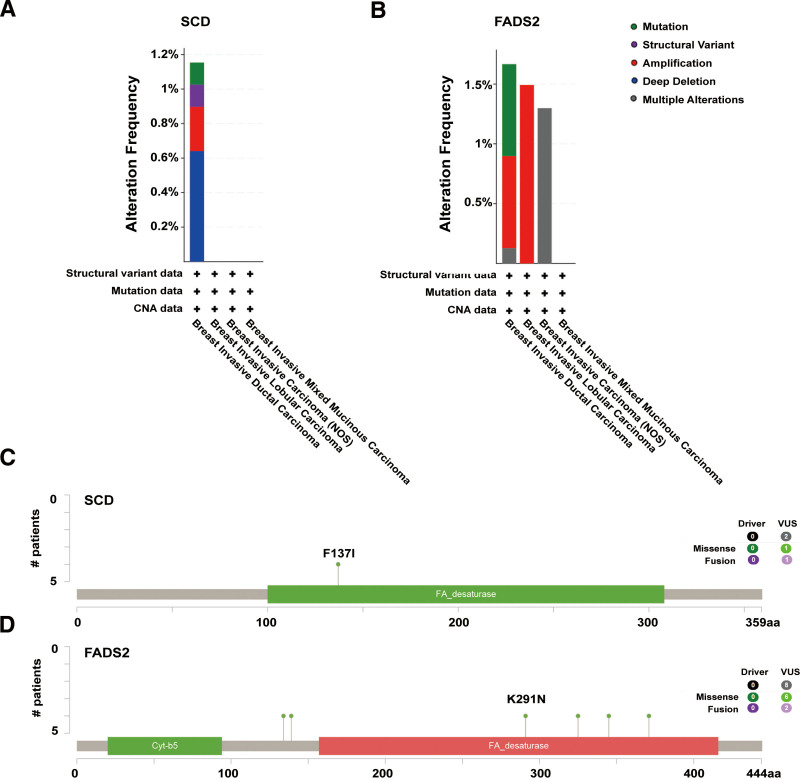
The genetic alterations of *SCD* or *FADS2* in BRCA of TCGA. Alteration summary of *SCD* (A) and *FADS2* (B) for BRCA tumors. The sites of *SCD* (C) or *FADS2* (D) genetic alterations using the cBioPortal online platform.

Furthermore, we investigated the genetic alteration characteristics of *SCD* and *FADS2* in the TCGA-BRCA cohort using the cBioPortal network. The primary types of alterations in *SCD* included “deep deletion”, “amplification”, “structural variant”, and “mutation” in patients with BRCA (Fig. 4A). For *FADS2*, the main alterations were “amplification”, “mutation”, and “multiple alterations” (Fig. 4B). The critical change in function was a mutation occurring at the active site of SCD or FADS2 desaturase, and missense or fusion mutations were the predominant mutations in *SCD* or *FADS2* mutation. For instance, a missense mutation within the F137I domain was found in *SCD* patients (Fig. 4C). and the K291N domain in *FADS2* (Fig. 4D) could be detected in BRCA cases.

### 3.5. Analysis of FA desaturase genes and immune infiltration in BRCA

**Figure 5.**
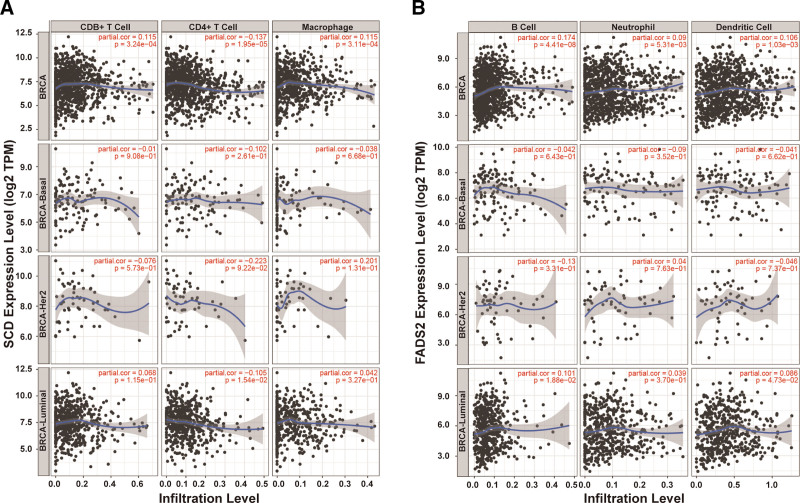
Correlation between *SCD* or *FADS2* expression and immune infiltrating cells. Relationship between *SCD* (A) or *FADS2* (B) expression levels and immune cell infiltration levels in BRCA, BRCA-basal, BRCA-Her2, and BRCA-luminal using the TIMER tool.

Recent studies have shown that immune cell infiltration is associated with the initiation, progression, and metastasis of human cancers. To further clarify the BRCA microenvironment and the mechanism by which FA desaturase affects BRCA heterogeneity and prognosis, we investigated whether *SCD or FADS2* is related to immune infiltration. The association between *SCD* or *FADS2* expression and tumor-infiltrating immune cells (B cells, CD8+ T cells, CD4+ T cells, macrophages, neutrophils, and dendritic cells) in BRCA was determined using the TIMER tool. The results showed that *SCD* expression was positively associated with CD4+ T cells and macrophages (Fig. 5A), and *FADS2* was positively correlated with B cell, neutrophil, and dendritic cell infiltration (Fig. 5B) in BRCA. We then focused on *SCD* (Table 2) and *FADS2* (Table 3) in BRCA with the immune cell markers of B cells, T cells, CD8+ T cells, monocytes, M1 macrophages, M2 macrophages, TAM, neutrophils, natural killer cells, dendritic cells, and functional T cell markers of Th1, Th2, Tfh, Th17, Treg, and T cell exhaustion. Notably, there were significant differences in the level of immune cell infiltration between the different groups based on *SCD* or *FADS2* (Fig. S4, Supplemental Digital Content, http://links.lww.com/MD/M968) copy numbers. In summary, the above results suggest that both *SCD* and *FADS2* expression could affect the tumor immune microenvironment by regulating immune cell infiltration.

**Table 2:** Correlation analysis between stearoyl-CoA desaturase 1 and related genes and markers of immune cells in BRCA.

Description	Gene markers	None		Purity	
		Cor	*P*	Cor	*P*
CD8+ T cell	*CD8A*	‐0.08744	0.003704	‐0.07891	0.012775
	*CD8B*	‐0.17922	2.15 e‐09	‐0.16838	9.14 e‐08
T cell (general)	*CD3D*	‐0.13966	3.32 e‐06	‐0.13193	2.98 e‐05
	*CD3E*	‐0.10085	0.000809	‐0.09121	0.003985
	*CD2*	‐0.07642	0.011236	‐0.06308	0.046695
B cell	*CD19*	‐0.11891	7.70 e‐05	‐0.11328	0.000343
	*CD79A*	‐0.11333	0.000166	‐0.11207	0.000397
Monocyte	*CD86*	0.07176	0.017295	0.074834	0.018231
	*CSF1R*	‐0.05884	0.051043	‐0.06562	0.03851
TAM	*CCL2*	‐0.03133	0.299156	‐0.03122	0.325207
	*CD68*	0.128177	2.01 e‐05	0.127394	5.58 e‐05
	*IL10*	0.095382	0.00154	0.104721	0.000939
M1 macrophage	*NOS2*	‐0.04511	0.134834	‐0.04965	0.117548
	*IRF5*	‐0.00377	0.900731	‐0.01599	0.614437
	*PTGS2*	‐0.16281	5.63 e‐08	‐0.15139	1.61 e‐06
M2 macrophage	*CD163*	0.117318	9.62 e‐05	0.115494	0.000261
	*VSIG4*	0.049067	0.103847	0.032893	0.299944
	*MS4A4A*	0.088948	0.003152	0.081953	0.009705
Neutrophils	*CEACAM8*	‐0.07559	0.012153	‐0.06853	0.030644
	*ITGAM*	‐0.0027	0.928818	‐0.00563	0.859196
	*CCR7*	‐0.10298	0.000625	‐0.08641	0.006382
Natural killer cell	*KIR2DL1*	‐0.02522	0.403388	‐0.0258	0.416233
	*KIR2DL3*	0.057244	0.057702	0.069965	0.027323
	*KIR2DL4*	‐0.00632	0.834247	0.005572	0.860659
	*KIR3DL1*	0.017923	0.552638	0.033634	0.289186
	*KIR3DL2*	‐0.02987	0.322318	‐0.01964	0.536048
	*KIR3DL3*	0.015	0.61922	0.014884	0.63913
	*KIR2DS4*	0.034518	0.252677	0.040178	0.205412
Dendritic cell	*HLA-DPB1*	‐0.17292	8.27 e‐09	‐0.17104	5.97 e‐08
	*HLA-DQB1*	‐0.12833	1.99 e‐05	‐0.11823	0.000187
	*HLA-DRA*	‐0.03225	0.285181	‐0.02375	0.454222
	*HLA-DPA1*	‐0.05303	0.078754	‐0.04943	0.119153
	*CD1C*	‐0.1949	7.07 e‐11	‐0.19143	1.15 e‐09
Th1	*NRP1*	0.084554	0.005013	0.08007	0.011518
	*ITGAX*	0.027039	0.370299	0.028221	0.373872
	*TBX21*	‐0.12185	5.08 e‐05	‐0.11303	0.000354
	*STAT1*	0.183654	8.44 e‐10	0.201084	1.55 e‐10
	*IFNG*	‐0.04337	0.150586	‐0.03314	0.296339
	*TNF*	‐0.06407	0.033608	‐0.04359	0.169463
Th2	*GATA3*	0.126461	2.60 e‐05	0.119517	0.000158
	*STAT6*	‐0.11191	0.0002	‐0.11216	0.000393
	*STAT5A*	‐0.20465	7.27 e‐12	‐0.19932	2.25 e‐10
	*IL13*	‐0.07021	0.019859	‐0.06077	0.055333
Tfh	*BCL6*	0.047248	0.117318	0.053571	0.091234
	*IL21*	0.03168	0.293828	0.061052	0.054209
Th17	*STAT3*	0.072044	0.016857	0.080486	0.01111
	*IL17A*	‐0.01155	0.701976	‐0.0231	0.466635
Treg	*FOXP3*	0.022783	0.450332	0.039602	0.211988
	*CCR8*	0.183835	8.12 e‐10	0.203741	8.77 e‐11
	*STAT5B*	‐0.01188	0.693863	‐0.01095	0.730184
	*TGFB1*	‐0.07241	0.016304	‐0.08016	0.011425
T cell exhaustion	*PDCD1*	‐0.1432	1.85 e‐06	‐0.13577	1.73 e‐05
	*CTLA4*	‐0.0205	0.49711	‐0.00586	0.853547
	*LAG3*	‐0.08386	0.005384	‐0.06969	0.027942
	*HAVCR2*	0.102615	0.000653	0.10004	0.00158
	*GZMB*	‐0.05939	0.048914	‐0.04759	0.133571

**Table 3:** Correlation analysis between fatty acid desaturase 2 and related genes and markers of immune cells in BRCA.

Description	Gene markers	None		Purity	
		Cor	*P*	Cor	*P*
CD8+ T cell	*CD8A*	‐0.01706	0.571969	‐0.01407	0.657589
	*CD8B*	0.022135	0.463324	0.024786	0.434815
T cell (general)	*CD3D*	0.010223	0.734861	0.008164	0.797033
	*CD3E*	0.026683	0.376621	0.02653	0.403177
	*CD2*	0.0474	0.116145	0.047445	0.134772
B cell	*CD19*	0.001669	0.955914	0.00777	0.806627
	*CD79A*	0.005468	0.856262	0.006244	0.844041
Monocyte	*CD86*	0.140492	2.90 e‐06	0.140467	8.69 e‐06
	*CSF1R*	0.015678	0.603471	0.013004	0.682038
TAM	*CCL2*	0.060611	0.044452	0.064158	0.043042
	*CD68*	0.133266	9.22 e‐06	0.132756	2.66 e‐05
	*IL10*	0.114362	0.000144	0.128288	4.94 e‐05
M1 macrophage	*NOS2*	0.045803	0.128966	0.039871	0.208906
	*IRF5*	0.020768	0.491399	0.028516	0.368893
	*PTGS2*	‐0.02036	0.50001	‐0.00308	0.922633
M2 macrophage	*CD163*	0.172868	7.91 e‐09	0.171558	5.18 e- 08
	*VSIG4*	0.078559	0.009145	0.071441	0.024224
	*MS4A4A*	0.100749	0.000819	0.098898	0.001788
Neutrophils	*CEACAM8*	0.019768	0.512504	0.012165	0.701526
	*ITGAM*	0.084305	0.005144	0.080654	0.010925
	*CCR7*	‐0.00732	0.808273	‐0.00278	0.930293
Natural killer cell	*KIR2DL1*	0.091169	0.002473	0.094523	0.00284
	*KIR2DL3*	0.155921	2.02 e‐07	0.159577	4.20 e‐07
	*KIR2DL4*	0.152515	3.73 e‐07	0.152103	1.44 e‐06
	*KIR3DL1*	0.139312	3.52 e‐06	0.145881	3.83 e‐06
	*KIR3DL2*	0.072544	0.016109	0.063867	0.043998
	*KIR3DL3*	0.103622	0.000577	0.091161	0.004003
	*KIR2DS4*	0.104523	0.000516	0.103525	0.001075
Dendritic cell	*HLA-DPB1*	‐0.05107	0.09046	‐0.04842	0.126937
	*HLA-DQB1*	0.009665	0.748821	0.011455	0.718183
	*HLA-DRA*	0.055374	0.066378	0.063939	0.043759
	*HLA-DPA1*	0.011628	0.700055	0.014907	0.638598
	*CD1C*	‐0.10759	0.000351	‐0.11577	0.000253
Th1	*NRP1*	0.028065	0.352408	0.033182	0.295726
	*ITGAX*	0.074644	0.013275	0.073482	0.020443
	*TBX21*	0.023043	0.44517	0.021096	0.506258
	*STAT1*	0.215519	5.01 e‐13	0.219874	2.33 e‐12
	*IFNG*	0.106924	0.000382	0.110707	0.000468
	*TNF*	0.12187	5.07 e‐05	0.117732	0.000198
Th2	*GATA3*	‐0.22426	5.26 e‐14	‐0.23567	5.04 e‐14
	*STAT6*	‐0.20406	8.37 e‐12	‐0.19377	7.13 e‐10
	*STAT5A*	‐0.11332	0.000166	‐0.11583	0.000251
	*IL13*	0.04085	0.175784	0.039752	0.210258
Tfh	*BCL6*	‐0.01442	0.632868	‐0.01596	0.615155
	*IL21*	0.135445	6.55 e‐06	0.147299	3.07 e‐06
Th17	*STAT3*	0.083049	0.00585	0.088121	0.005409
	*IL17A*	0.082224	0.006361	0.075166	0.017721
Treg	*FOXP3*	0.173795	6.56 e‐09	0.172894	4.06 e‐08
	*CCR8*	0.215455	5.10 e‐13	0.215902	5.86 e‐12
	*STAT5B*	‐0.11165	0.000207	‐0.10543	0.000866
	*TGFB1*	‐0.10296	0.000626	‐0.10623	0.00079
T cell exhaustion	*PDCD1*	0.032612	0.279848	0.032056	0.312419
	*CTLA4*	0.148465	7.58 e‐07	0.150479	1.86 e‐06
	*LAG3*	0.136144	5.86 e‐06	0.139334	1.03 e‐05
	*HAVCR2*	0.113	0.000173	0.111076	0.000448
	*GZMB*	0.153589	3.08 e‐07	0.153864	1.08 e‐06

### 3.6. Single-cell expression levels of FA desaturase genes in BRCA cancer tissues

**Figure 6.**
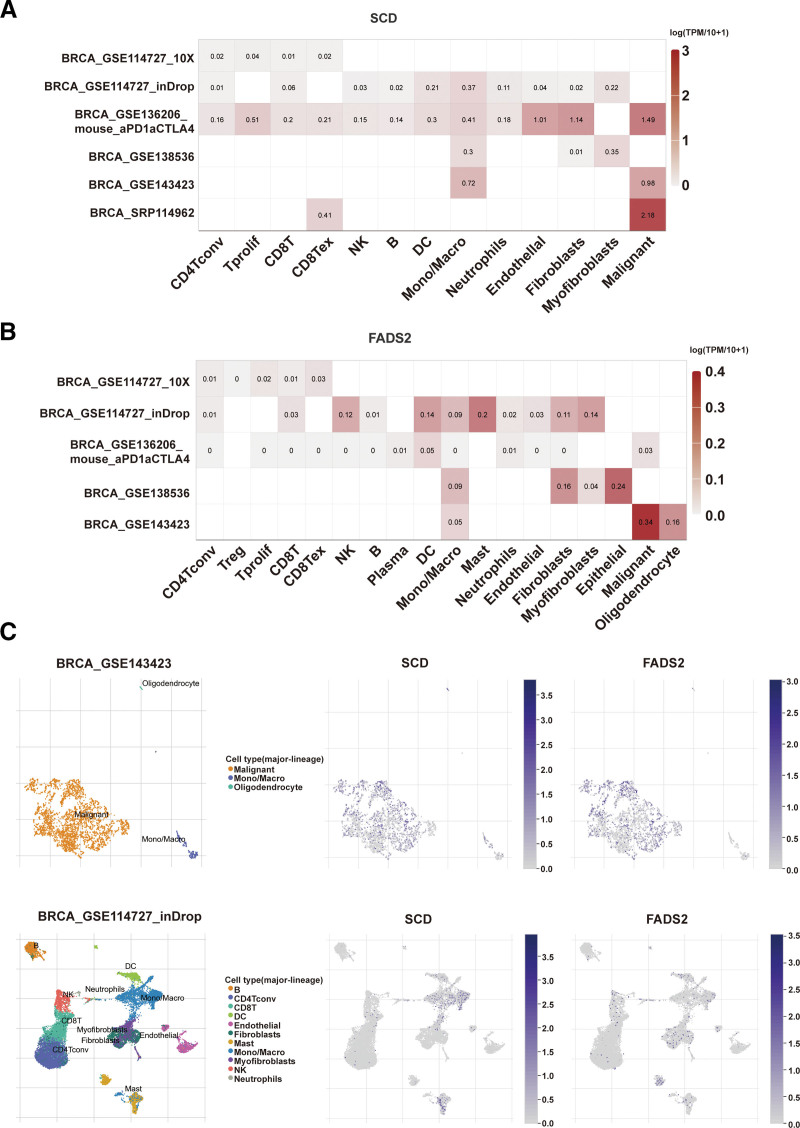
The expression levels of *SCD* and *FADS2* at single-cell levels. The heatmap displays the average expression of *SCD* (A) and *FADS2* (B) in TME-related cells. (C) Distribution of *SCD* or *FADS2* at single-cell resolution in BRCA_GSE143423 and BRCA_GSE114727_inDrop datasets across TISCH tool.

To study the potential role of FA desaturases in the immunity of BRCA, the TISCH database was used to explore *SCD* and *FADS2* expression in the TME. As for other components of the TME, we found that *SCD* was mainly overexpressed in malignant tumor cells, endothelial cells, fibroblast cells, and macrophage cells, whereas it was relatively low for the immune cell components of CD4 Tconv cells, natural killer cells, B cells, and neutrophils (Fig. 6A). Meanwhile, the *FADS2* expression level was mainly upregulated in malignant tumor cells, epithelial cells, mast cells, and macrophages, whereas it was extremely low in CD4 Tconv cells, Treg cells, plasma cells, and neutrophils (Fig. 6B). The distribution of *SCD* or *FADS2* expression from each dataset is shown in Figure 6C, the UMAP plots showed that *SCD* and *FADS2* expression levels remained higher in malignant tumor cells of the BRCA_GSE143423 dataset and macrophages and mast cells of the BRCA_GSE114727_inDrop dataset. These findings suggest that, in addition to malignant cells, *SCD* and *FADS2* played a role in immune cells and stromal cells.

### 3.7. PPI network and potential functional status of FA desaturase genes

**Figure 7.**
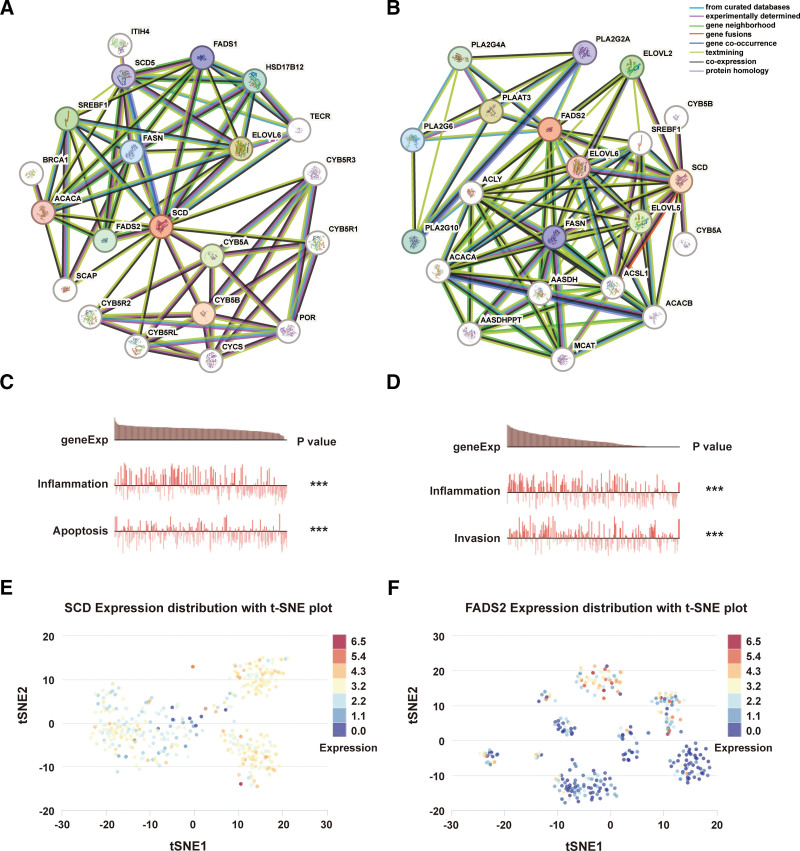
PPI network and cancer functional states of *SCD* or *FADS2*. A network of *SCD* (A) and FADS2 (B) co-expressed genes. Functional relevance of *SCD* (C) and *FADS2* (D) in EXP0052 from TCGA-BRCA. *SCD* (E) and *FADS2* (F) expression profiles are shown at single-cell levels from TCGA-BRCA by T-SNE plot via the CancerSEA website (*P < 0.05; **P < 0.01; ***P < 0.001).

A protein interaction network is necessary for understanding the molecular mechanisms of malignancy. To explore the potential molecular mechanisms of SCD and FADS2, we conducted network analysis of FA desaturases using the STRING tool. The results illustrated a co-expressed protein network of *SCD* (Fig. 7A) or *FADS2* (Fig. 7B) interacting genes in the PPI networks. Among them, sterol regulatory element-binding transcription factor 1, fatty acid synthase, acetyl-CoA carboxylase alpha, and cytochrome b5 type A are common interactors of *SCD* and *FADS2*, which provides clues for further study of their functions. In addition, single-cell transcriptome sequencing is a key technique for analyzing the potential functions of candidate molecules at the single-cell level.^[39]^ CancerSEA was used to determine whether the module is associated with carcinogenic processes in different subpopulations of BRCA cells. The results showed that *SCD* was correlated with inflammation and apoptosis (Fig. 7C), and *FADS2* was correlated with inflammation and DNA repair (Fig. 7D). The module could promote BRCA by splicing and processing mRNA based on the clustering results of CancerSEA, and the overall expressions of *SCD* (Fig. 7E) and *FADS2* (Fig. 7F) in BRCA were found to be heterogeneous.

### 3.8. Effect of FA desaturase genes on BRCA cell proliferation

**Figure 8.**
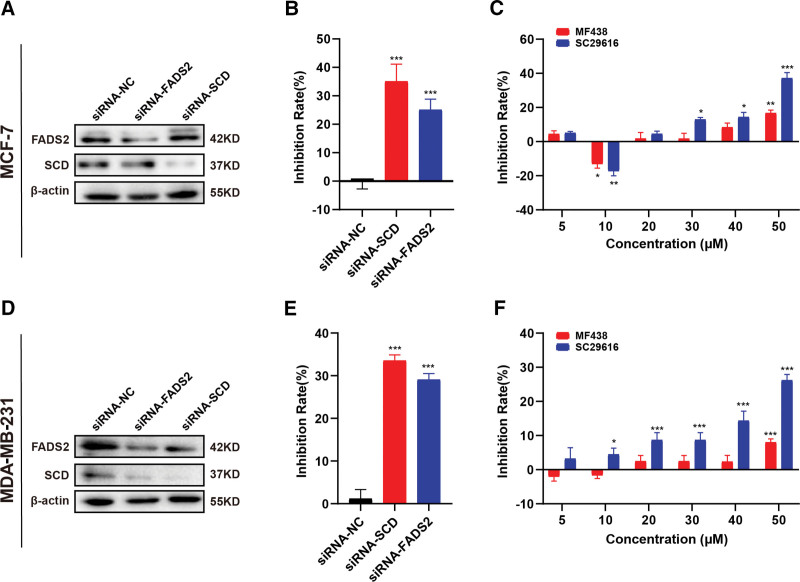
*SCD* and *FADS2* affects proliferation of breast cancer cells. The expression of SCD, FADS2, and β-actin was tested by western blotting in MCF-7 (A) and MDA-MB-231 (D) cells. Knockdown of siRNA and inhibitors reduced proliferation of MCF-7 cells (B and C) and MDA-MB-231 cells (E and F) by MTT assays (n = 3, *P < 0.05; **P < 0.01; ***P < 0.001).

To verify the efficacy of FA desaturases on BRCA cell proliferation, we used siRNA to knock down *SCD* or *FADS2* in MCF-7 and MDA-MB-231 cells. Compared with the silencing negative control RNA interference cells, SCD or FADS2 knockdown cells showed a significant decrease in SCD or FADS2 protein expression, indicating successful knockdown (Fig. 8A and D) and the inhibition of BRCA cell proliferation was manifested (Fig. 8B and E). Simultaneously, there was a minimal inhibitory effect on the growth of either cell line at a low dose of SCD inhibitor MF438 and FADS2 inhibitor A939572, while significant inhibiting effect on growth when using MF438 and A939572 over 50 μM (Fig. 8C and F). These results show that both SCD and FADS2 had a significant impact on BRCA cell proliferation.

## 4. Discussion

Compared with normal cells, the metabolic characteristics of cancer cells undergo significant changes, including an abnormally high demand for glucose and glutamine, de novo FA synthesis, higher lipid production, and FA uptake. De novo FA synthesis involves carboxylation, addition, desaturation, and extension of the carbon chain of acetyl-CoA. Recent studies have found that FA desaturation is closely related to tumor development and plays a crucial role in determining the fate of tumors.^[14]^ Currently, FA desaturases include SCD and FADS2.^[40]^ Among FA desaturases, SCD is the most widely studied, and it promotes tumor initiation, occurrence, development, metastasis, and stemness formation.^[41,42]^ Consistent with the role of SCD in promoting tumor growth, our previous studies also found that a new SCD inhibitor could induce SCD-dependent apoptosis and autophagy in BRCA cells, which benefits breast cancer treatment.^[16,43]^ However, recent studies have confirmed that simply inhibiting SCD in tumor cells leads to an increase in FADS2 activity and promotes tumor growth instead.^[17]^ Therefore, it is important to explore the expression and significance of FA desaturases, represented by SCD and FADS2, in tumors and their role in the BRCA immune microenvironment, tumor diagnosis, and prognosis.

Based on multivariate analysis, *SCD* or *FADS2* was identified as a prognostic risk factor for 33 types of cancer, especially BRCA. In addition, both *SCD* and *FADS2* had the highest correlation with the molecular subtype of BRCA, especially Her2 positive BRCA. Further, UALCAN analysis of the correlation between *SCD* or *FADS2* expression and patient clinicopathological parameters showed that higher *SCD* and *FADS2* expression participated in BRCA progression. These data indicate that FA desaturation promotes BRCA development and further suggests the synergistic effect of *SCD* and *FADS2* on BRCA progression. In the future, we will continue to explore the role of FA desaturases in tumor diagnosis and prognosis using clinical data.

Methylation of the promoter CpG island blocks binding of the transcription factor complex to the promoter, thereby inhibiting gene expression.^[44]^ Hypermethylation often leads to transcriptional silencing of tumor suppressor genes and DNA repair genes, resulting in abnormal regulation of growth and differentiation of normal cells and failure to repair DNA damage, leading to the occurrence of tumors.^[45]^ Further analysis of the promoter methylation level between *SCD* or *FADS2* expression and patient clinicopathological parameters showed that *SCD* and *FADS2* methylation were significantly related to major subclasses, patient sex, age, race, nodal metastasis status, tumor history, menopause status, and individual cancer stages. Additionally, the data suggested that the “deep deletion” type was the predominant alteration for *SCD*, while the “amplification” type was the main alteration for *FADS2* in BRCA. In 8 BRCA cases, a missense mutation within the F137I domain in *SCD* and the K291N domain in *FADS2* was also detected.

The functional roles of *SCD* and *FADS2* showed that *SCD* is correlated with inflammation and apoptosis, whereas *FADS2* is correlated with inflammation and DNA repair. The difference in cell signaling between *SCD* and *FADS2* might be due to inherent heterogeneity in BRCA or the limited number of samples for single-cell scRNA-Seq analysis. To further investigate the potential molecular mechanisms of *SCD* and *FADS2*, the STRING database was used to predict protein-protein interactions. The results showed that sterol regulatory element-binding transcription factor 1, fatty acid synthase, acetyl-CoA carboxylase alpha, and cytochrome b5 type A were predicted to be functional partners of *SCD* and *FADS2*, which provided clues for FA metabolism cell signaling in BRCA.

During the formation and growth of the tumor, immune cells not only suppress the tumor but also shape the immunogenicity of cancer cells in the elimination, equilibrium, and escape phase.^[46]^ Therefore, immune cells in the TME include natural killer cells, macrophages, neutrophils, B cells, and T cells, which play pivotal roles in BRCA onset, development, metastasis, and spread.^[47]^ Tumor-associated macrophages also support cancer cell growth and metastasis and mediate immunosuppressive effects on adaptive immune cells in the TME.^[48–50]^ In our study, we found that *SCD* and *FADS2* expression were significantly positively associated with macrophage cells. Therefore, SCD and FADS2 could be used as potential targets to regulate the immune microenvironment, thereby regulating the occurrence and development of tumors.

However, there are still some limitations to present study. Firstly, the animal experiments in vivo are needed to further verify the anti-proliferation effect of SCD1 or FADS2 inhibitors on the BRCA cells. Secondly, considering the promoting effect of SCD1 and FADS2 on breast cancer development, how to combine these inhibitors to promote the therapeutic effect of existing chemotherapy drugs is a question worth exploring. Finally, some omics techniques such as transcriptomics, genomics, and metabolomics are needed to investigate how SCD1or FADS2 regulates related cell signaling and metabolic change to accelerate BRCA development.

## 5. Conclusions

Overall, this study provides an in-depth understanding of the heterogeneity and complexity of the molecular biological characteristics of *SCD* and *FADS2* by analyzing the prognosis, mutations, and tumor immune microenvironment in BRCA. We demonstrated that SCD1 and FADS2 could be potential biomarkers to predict or diagnose aggressive breast cancer, and the SCD1 or FADS2 expression is positively related to infiltration of immune cells in tumor microenvironment.

## Author contributions

**Conceptualization:** Guosheng Wu.

**Data curation:** Jie Wang, Qian Zhang.

**Formal analysis:** Qian Zhang.

**Funding acquisition:** Zhangfeng Zhong.

**Investigation:** Yixuan Wang, Guosheng Wu.

**Methodology:** Jie Wang.

**Project administration:** Qian Zhang, Duanrui Zhou, Zhangfeng Zhong, Guosheng Wu.

**Resources:** Duanrui Zhou.

**Software:** Yixuan Wang.

**Supervision:** Huilian Che, Yunjun Ge, Zhangfeng Zhong.

**Validation:** Yixuan Wang, Huilian Che, Yunjun Ge.

**Visualization:** Yunjun Ge.

**Writing – original draft:** Jie Wang.

**Writing – review & editing:** Guosheng Wu, Zhangfeng Zhong.

## Supplementary Material









## References

[1] Bray F Ferlay J Soerjomataram I Siegel RL Torre LA Jemal A. Global cancer statistics 2018: GLOBOCAN estimates of incidence and mortality worldwide for 36 cancers in 185 countries. CA Cancer J Clin. 2018;68:394–424. 30207593 10.3322/caac.21492

[2] Sung H Ferlay J Siegel RL. Global cancer statistics 2020: GLOBOCAN estimates of incidence and mortality worldwide for 36 cancers in 185 countries. CA Cancer J Clin. 2021;71:209–49. 33538338 10.3322/caac.21660

[3] Di Leo A Curigliano G Diéras V. New approaches for improving outcomes in breast cancer in Europe. Breast. 2015;24:321–30. 25840656 10.1016/j.breast.2015.03.001

[4] Maruthanila VL Elancheran R Kunnumakkara AB Kabilan S Kotoky J. Recent development of targeted approaches for the treatment of breast cancer. Breast Cancer. 2017;24:191–219. 27796923 10.1007/s12282-016-0732-1

[5] Esteva FJ Hubbard-Lucey VM Tang J Pusztai L. Immunotherapy and targeted therapy combinations in metastatic breast cancer. Lancet Oncol. 2019;20:e175–86. 30842061 10.1016/S1470-2045(19)30026-9

[6] Ookhtens M Kannan R Lyon I Baker N. Liver and adipose tissue contributions to newly formed fatty acids in an ascites tumor. Am J Physiol. 1984;247:R146–53. 6742224 10.1152/ajpregu.1984.247.1.R146

[7] Fritz V Benfodda Z Rodier G. Abrogation of de novo lipogenesis by stearoyl-CoA desaturase 1 inhibition interferes with oncogenic signaling and blocks prostate cancer progression in mice. Mol Cancer Ther. 2010;9:1740–54. 20530718 10.1158/1535-7163.MCT-09-1064 PMC3315476

[8] Agostini M Almeida LY Bastos DC. The fatty acid synthase inhibitor orlistat reduces the growth and metastasis of orthotopic tongue oral squamous cell carcinomas. Mol Cancer Ther. 2014;13:585–95. 24362464 10.1158/1535-7163.MCT-12-1136

[9] Chen M Zhang R Chen Y. Nobiletin inhibits de novo FA synthesis to alleviate gastric cancer progression by regulating endoplasmic reticulum stress. Phytomedicine. 2023;116:154902. 37270969 10.1016/j.phymed.2023.154902

[10] Pavlova NN Zhu J Thompson CB. The hallmarks of cancer metabolism: Still emerging. Cell Metab. 2022;34:355–77. 35123658 10.1016/j.cmet.2022.01.007 PMC8891094

[11] Tsai YW Lu C-H Chang RC Hsu Y-P Ho L-T Shih K-C. Palmitoleic acid ameliorates palmitic acid-induced proinflammation in J774A.1 macrophages via TLR4-dependent and TNF-α-independent signallings. Prostaglandins Leukot Essent Fatty Acids. 2021;169:102270. 33930845 10.1016/j.plefa.2021.102270

[12] Peck B Schulze A. Lipid desaturation-the next step in targeting lipogenesis in cancer? FEBS J. 2016;283:2767–78. 26881388 10.1111/febs.13681

[13] Igal RA. Stearoyl-CoA desaturase-1: a novel key player in the mechanisms of cell proliferation, programmed cell death and transformation to cancer. Carcinogenesis. 2010;31:1509–15. 20595235 10.1093/carcin/bgq131

[14] Zhao G Tan Y Cardenas H. Ovarian cancer cell fate regulation by the dynamics between saturated and unsaturated fatty acids. Proc Natl Acad Sci U S A. 2022;119:e2203480119. 36197994 10.1073/pnas.2203480119 PMC9564215

[15] Min JY Kim DH. Stearoyl-CoA desaturase 1 as a therapeutic biomarker: focusing on cancer stem cells. Int J Mol Sci. 2023;24:8951. 37240297 10.3390/ijms24108951 PMC10219200

[16] Yang C Jin Y-Y Mei J. Identification of icaritin derivative IC2 as an SCD-1 inhibitor with anti-breast cancer properties through induction of cell apoptosis. Cancer Cell Int. 2022;22:202. 35642041 10.1186/s12935-022-02621-y PMC9153146

[17] Vriens K Christen S Parik S. Evidence for an alternative fatty acid desaturation pathway increasing cancer plasticity. Nature. 2019;566:403–6. 30728499 10.1038/s41586-019-0904-1 PMC6390935

[18] Simopoulos AP. Genetic variants in the metabolism of omega-6 and omega-3 fatty acids: their role in the determination of nutritional requirements and chronic disease risk. Exp Biol Med (Maywood). 2010;235:785–95. 20558833 10.1258/ebm.2010.009298

[19] D’Andrea S Guillou H Jan S. The same rat Delta6-desaturase not only acts on 18- but also on 24-carbon fatty acids in very-long-chain polyunsaturated fatty acid biosynthesis. Biochem J. 2002;364:49–55. 11988075 10.1042/bj3640049 PMC1222544

[20] Cho HP Nakamura MT Clarke SD. Cloning, expression, and nutritional regulation of the mammalian Delta-6 desaturase. J Biol Chem. 1999;274:471–7. 9867867 10.1074/jbc.274.1.471

[21] He C Qu X Wan J. Inhibiting delta-6 desaturase activity suppresses tumor growth in mice. PLoS One. 2012;7:e47567. 23112819 10.1371/journal.pone.0047567 PMC3480421

[22] Jiang Y Mao C Yang R. EGLN1/c-Myc induced lymphoid-specific helicase inhibits ferroptosis through lipid metabolic gene expression changes. Theranostics. 2017;7:3293–305. 28900510 10.7150/thno.19988 PMC5595132

[23] Xuan Y Wang H Yung MM. SCD1/FADS2 fatty acid desaturases equipoise lipid metabolic activity and redox-driven ferroptosis in ascites-derived ovarian cancer cells. Theranostics. 2022;12:3534–52. 35547771 10.7150/thno.70194 PMC9065188

[24] Zhao T Gao P Li Y. Investigating the role of FADS family members in breast cancer based on bioinformatic analysis and experimental validation. Front Immunol. 2023;14:1074242. 37122728 10.3389/fimmu.2023.1074242 PMC10130515

[25] Tang Z Li C Kang B Gao G Li C Zhang Z. GEPIA: a web server for cancer and normal gene expression profiling and interactive analyses. Nucleic Acids Res. 2017;45:W98–W102. 28407145 10.1093/nar/gkx247 PMC5570223

[26] Mizuno H Kitada K Nakai K. PrognoScan: a new database for meta-analysis of the prognostic value of genes. BMC Med Genomics. 2009;2:18. 19393097 10.1186/1755-8794-2-18 PMC2689870

[27] Li T Fan J Wang B. TIMER: a web server for comprehensive analysis of tumor-infiltrating immune cells. Cancer Res. 2017;77:e108–10. 29092952 10.1158/0008-5472.CAN-17-0307 PMC6042652

[28] Ru B Wong CN Tong Y. TISIDB: an integrated repository portal for tumor-immune system interactions. Bioinformatics. 2019;35:4200–2. 30903160 10.1093/bioinformatics/btz210

[29] Chandrashekar DS Karthikeyan SK Korla PK. UALCAN: an update to the integrated cancer data analysis platform. Neoplasia. 2022;25:18–27. 35078134 10.1016/j.neo.2022.01.001 PMC8788199

[30] Cerami E Gao J Dogrusoz U. The cBio cancer genomics portal: an open platform for exploring multidimensional cancer genomics data. Cancer Discov. 2012;2:401–4. 22588877 10.1158/2159-8290.CD-12-0095 PMC3956037

[31] Gao J Aksoy BA Dogrusoz U. Integrative analysis of complex cancer genomics and clinical profiles using the cBioPortal. Sci Signal. 2013;6:pl1. 23550210 10.1126/scisignal.2004088 PMC4160307

[32] Sun D Wang J Han Y. TISCH: a comprehensive web resource enabling interactive single-cell transcriptome visualization of tumor microenvironment. Nucleic Acids Res. 2021;49:D1420–30. 33179754 10.1093/nar/gkaa1020 PMC7778907

[33] Szklarczyk D Gable AL Lyon D. STRING v11: protein-protein association networks with increased coverage, supporting functional discovery in genome-wide experimental datasets. Nucleic Acids Res. 2019;47:D607–13. 30476243 10.1093/nar/gky1131 PMC6323986

[34] Yuan H Yan M Zhang G. CancerSEA: a cancer single-cell state atlas. Nucleic Acids Res. 2019;47:D900–8. 30329142 10.1093/nar/gky939 PMC6324047

[35] Polyak K Metzger Filho O. SnapShot: breast cancer. Cancer Cell. 2012;22:562–562.e1. 23079664 10.1016/j.ccr.2012.06.021

[36] Lambrechts Y Garg AD Floris G. Circulating biomarkers at diagnosis correlate with distant metastases of early luminal-like breast cancer. Genes Immun. 2023;24:270–9. 37759086 10.1038/s41435-023-00220-z PMC10575765

[37] Ades F Zardavas D Bozovic-Spasojevic I. Luminal B breast cancer: molecular characterization, clinical management, and future perspectives. J Clin Oncol. 2014;32:2794–803. 25049332 10.1200/JCO.2013.54.1870

[38] Smith J Sen S Weeks RJ Eccles MR Chatterjee A. Promoter DNA hypermethylation and paradoxical gene activation. Trends Cancer. 2020;6:392–406. 32348735 10.1016/j.trecan.2020.02.007

[39] He J Ding H Li H Pan Z Chen Q. Intra-tumoral expression of SLC7A11 is associated with immune microenvironment, drug resistance, and prognosis in cancers: a pan-cancer analysis. Front Genet. 2021;12:770857. 34938318 10.3389/fgene.2021.770857 PMC8687742

[40] Wang Z Park HG Wang DH Kitano R Kothapalli KSD Brenna JT. Fatty acid desaturase 2 (FADS2) but not FADS1 desaturates branched chain and odd chain saturated fatty acids. Biochim Biophys Acta Mol Cell Biol Lipids. 2020;1865:158572. 31751799 10.1016/j.bbalip.2019.158572

[41] Luo Y Huang S Wei J. Long noncoding RNA LINC01606 protects colon cancer cells from ferroptotic cell death and promotes stemness by SCD1-Wnt/β-catenin-TFE3 feedback loop signalling. Clin Transl Med. 2022;12:e752. 35485210 10.1002/ctm2.752 PMC9052012

[42] Rahimi Y Mehdizadeh A Nozad Charoudeh H. Hepatocyte differentiation of human induced pluripotent stem cells is modulated by stearoyl-CoA desaturase 1 activity. Dev Growth Differ. 2015;57:667–74. 26676854 10.1111/dgd.12255

[43] Wang YX Jin Y-Y Wang J. Icaritin derivative IC2 induces cytoprotective autophagy of breast cancer cells via SCD1 inhibition. Molecules. 2023;28:1109. 36770781 10.3390/molecules28031109 PMC9920188

[44] Zhu H Wang G Qian J. Transcription factors as readers and effectors of DNA methylation. Nat Rev Genet. 2016;17:551–65. 27479905 10.1038/nrg.2016.83 PMC5559737

[45] Xia L Huang W Bellani M. CHD4 has oncogenic functions in initiating and maintaining epigenetic suppression of multiple tumor suppressor genes. Cancer Cell. 2017;31:653–68.e7. 28486105 10.1016/j.ccell.2017.04.005 PMC5587180

[46] Mittal D Gubin MM Schreiber RD Smyth MJ. New insights into cancer immunoediting and its three component phases—elimination, equilibrium and escape. Curr Opin Immunol. 2014;27:16–25. 24531241 10.1016/j.coi.2014.01.004 PMC4388310

[47] Zarrilli G Businello G Dieci MV. The tumor microenvironment of primitive and metastatic breast cancer: implications for novel therapeutic strategies. Int J Mol Sci. 2020;21:8102. 33143050 10.3390/ijms21218102 PMC7662409

[48] Christofides A Strauss L Yeo A Cao C Charest A Boussiotis VA. The complex role of tumor-infiltrating macrophages. Nat Immunol. 2022;23:1148–56. 35879449 10.1038/s41590-022-01267-2 PMC10754321

[49] Yang J Liao D Chen C. Tumor-associated macrophages regulate murine breast cancer stem cells through a novel paracrine EGFR/Stat3/Sox-2 signaling pathway. Stem Cells. 2013;31:248–58. 23169551 10.1002/stem.1281

[50] Mou W Xu Y Ye Y. Expression of Sox2 in breast cancer cells promotes the recruitment of M2 macrophages to tumor microenvironment. Cancer Lett. 2015;358:115–23. 25444903 10.1016/j.canlet.2014.11.004

