# Supplementary figures and images for: Systematic analysis of fatty acid desaturases in breast invasive carcinoma: The prognosis, gene mutation, and tumor immune microenvironment
Source: Medicine (Baltimore). 2024 Jun 21;103(25):e38597. doi: 10.1097/MD.0000000000038597 (PMC11191958; doi:10.1097/MD.0000000000038597)

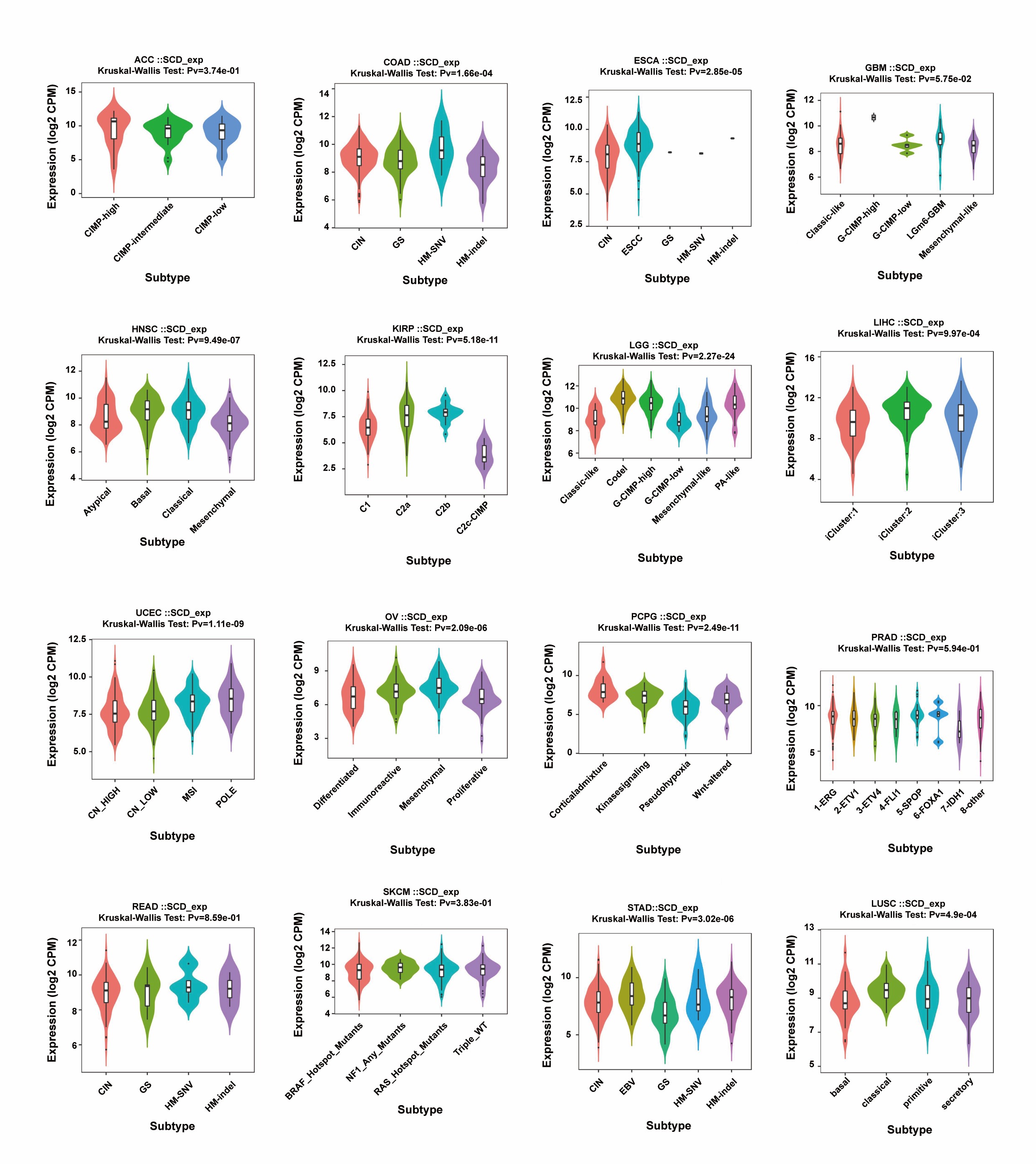

Supplement: Supplementary file 1 [file medi-103-e38597-s001.jpeg]

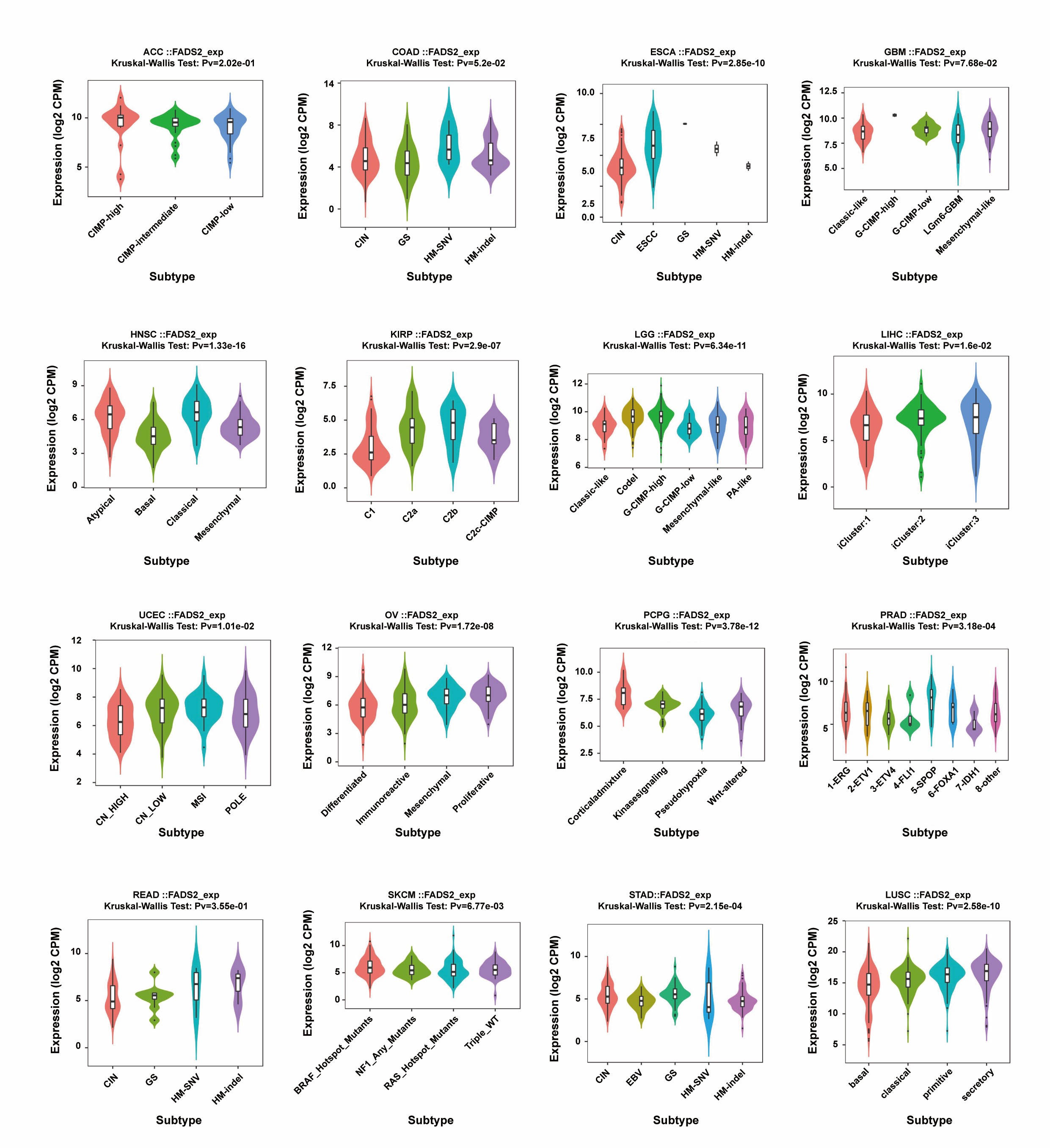

Supplement: Supplementary file 2 [file medi-103-e38597-s002.jpeg]

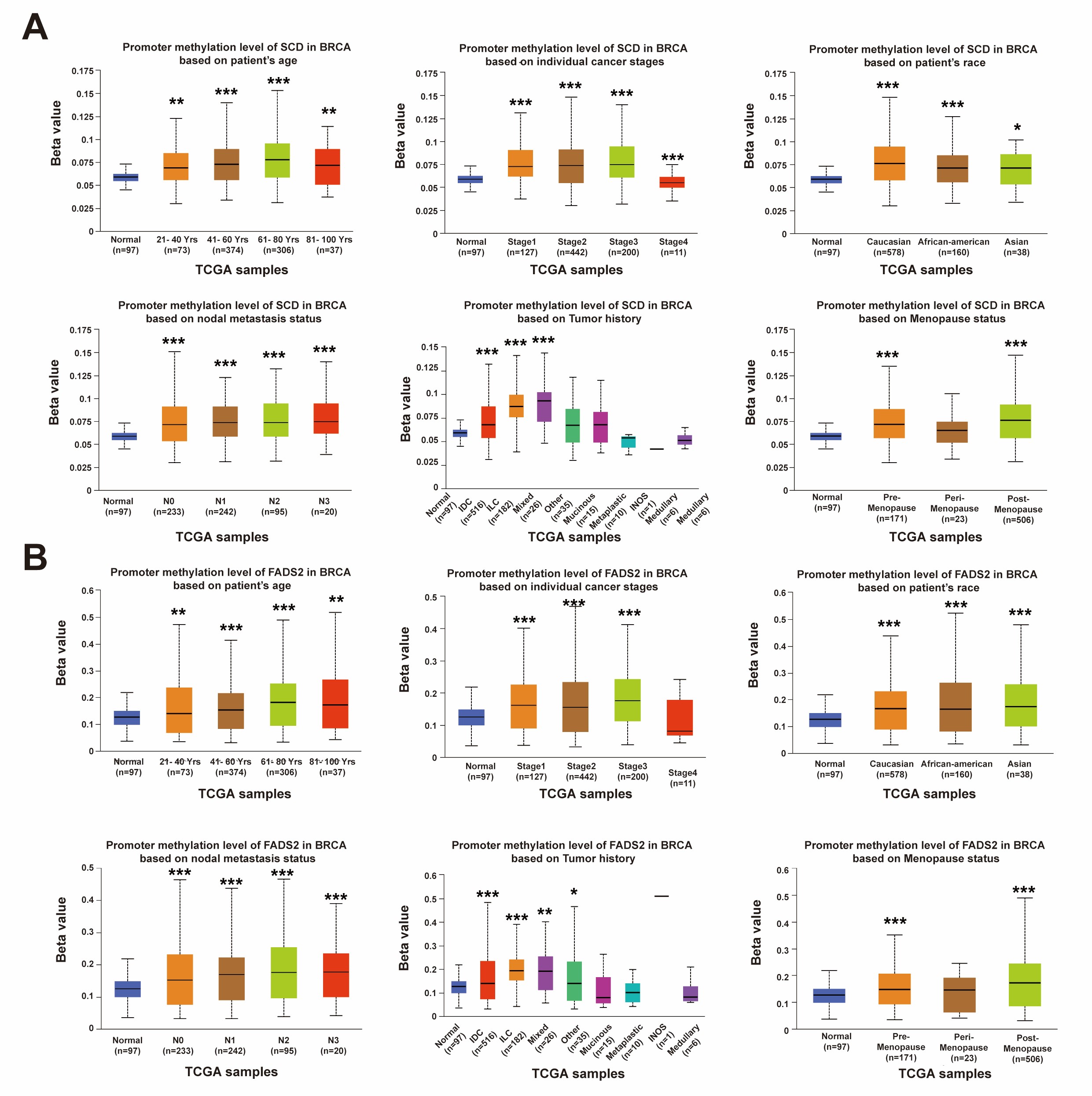

Supplement: Supplementary file 3 [file medi-103-e38597-s003.jpeg]

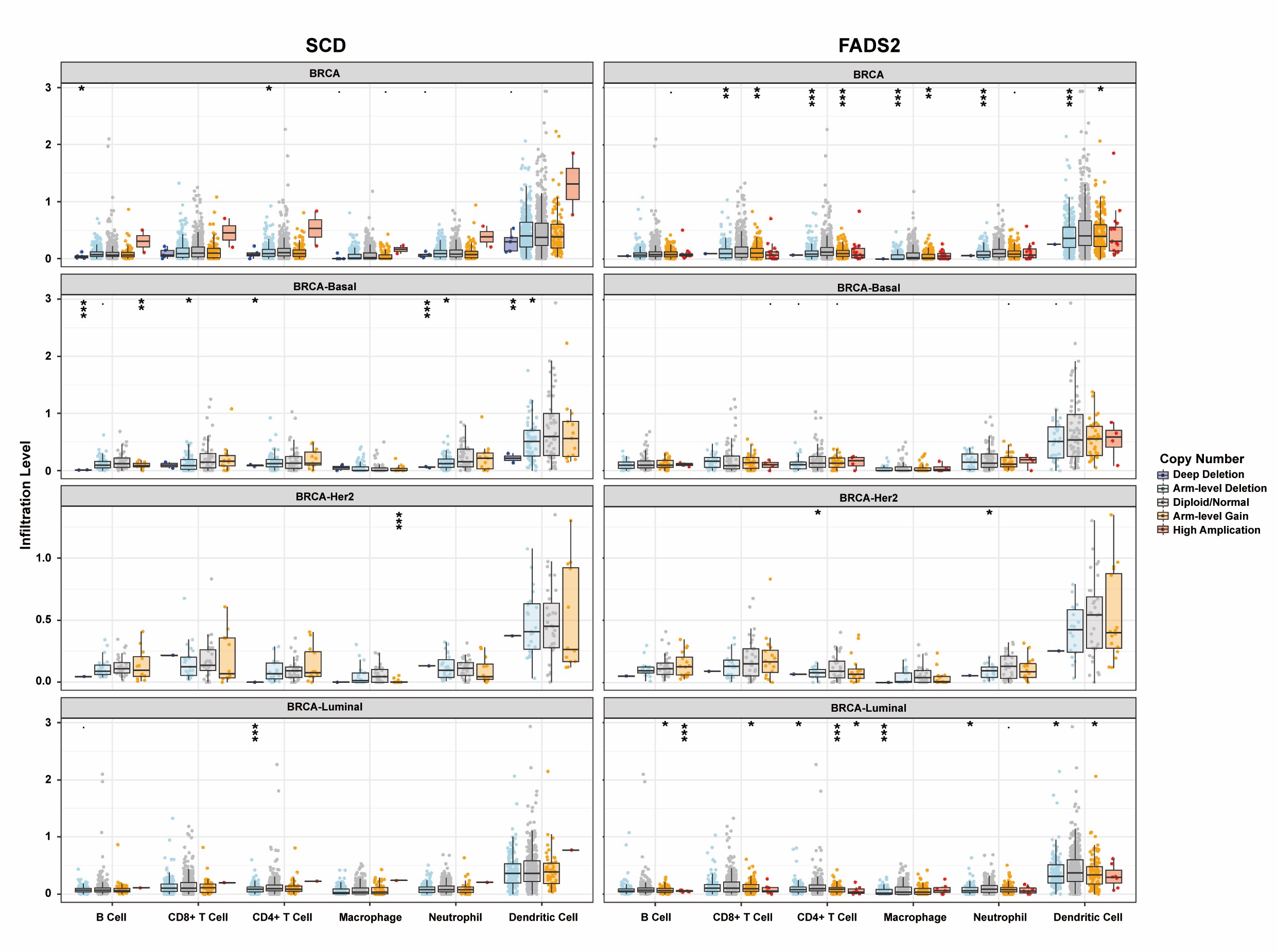

Supplement: Supplementary file 4 [file medi-103-e38597-s004.jpeg]
